# Supplementary material for: Healthcare workers´ perspectives on antibiotic utilization in children under five years of age in the Eastern Democratic Republic of the Congo
Source: Antimicrob Resist Infect Control. 2025 Jul 31;14:93. doi: 10.1186/s13756-025-01596-5 (PMC12315395; doi:10.1186/s13756-025-01596-5)
Supplement: Supplementary file 1 — Supplementary Material 1 [file 13756_2025_1596_MOESM1_ESM.pdf]

APPENDIX 1

INTERVIEW GUIDE FOR DOCTORS AND NURSES:

My name is………., my colleague’s name is…… we come from Panzi hospital and UEA, We are a research team on antibiotics. We have come to talk to the healtworkers who care for children under five in your institution. Our discussion will take about 40 minutes. If you don't mind, I'd like to take notes and record our discussion to make it easier for me to use later. I won't use your names.

- How old are you? :

- What is your profession? :

- How long have you worked as a doctor/nurse? :

- According to your experience, how long have you been working in paediatrics (treating children since ... or other categories of patients?):

Questions Probes:

1. How long have you been caring for children under the age of five?

2. What pathologies are frequently encountered in children under five in your hospital?

3. What medicines do you frequently used to treat children under the age of 5?

4. Can you tell me which drug you use to treat pathology X (pathology cited by the participant) and how?

5. What is your opinion on the use of antibiotics in children under 5 (what are the challenges?)? Do you have any advice on the use of antibiotics?)

6. Thank you for your participation

APPENDIX 2

INTERVIEW GUIDE FOR DRUG SELLERS:

My name is ……, my colleague's name is……We come from Panzi Hospital and UEA, We are a team of researchers on antibiotics. We have come to talk to the nursing staff who care for children under five in your institution. Our discussion will take about 40 minutes. If you don't mind, I'd like to take notes and record our discussion to make it easier for me to use later. I won't use your names.

- How old are you? :

- What is your profession? :

- How long have you worked as a pharmacist/nurse? :

- According to your experience, how long have you worked in a pharmacy (selling medicines for children since ... or other categories of patients?):

- Can you describe your experience with antibiotics?

Probes:

1. Do any mothers come to you to ask what medication to give their children, according to the symptoms they have specified?

2. What medications do you often use with children under 5?

3. Why these drugs? (See drug listed in question 2)

4. How do they know which medicines they want? (Probes: How often do they ask you for advice? Among the medicines you mentioned, are there any antibiotics?

5. Are there any antibiotics that you often serve? (What are your tips/opinions on the use of antibiotics?)

6. Thank you for your participation
