# Supplementary material for: Healthcare workers´ perspectives on antibiotic utilization in children under five years of age in the Eastern Democratic Republic of the Congo
Source: Antimicrob Resist Infect Control. 2025 Jul 31;14:93. doi: 10.1186/s13756-025-01596-5 (PMC12315395; doi:10.1186/s13756-025-01596-5)
Supplement: Supplementary file 2 — Supplementary Material 2 [file 13756_2025_1596_MOESM2_ESM.docx]

**Healthcare workers´ perspectives on antibiotic utilization in children under five years of age in the Eastern Democratic Republic of the Congo**

Jeannière T. Manegabe^1,2^, Rose Mwangi^3^, John Mulindwa^1,2^, David Sumaili^1^, Gloire M. Kapalata^1^, Rune Andersson^4,5,6^, Margret Lepp^8,9,10,11,12^, Florida Muro^3^, Susann Skovbjerg^4,5,6^, Matilda Emgård^4,6,7^, Archippe M. Birindwa^1,2^

1. Université Evangélique en Afrique, Bukavu/ Democratic Republic of Congo
2. Panzi Hospital, Paediatrics Department, Bukavu/ Democratic Republic of Congo
3. School of Public Health, KCMC University, Moshi, Tanzania
4. Department of Infectious Diseases, Institute of Biomedicine, Sahlgrenska Academy, University of Gothenburg, Gothenburg, Sweden
5. Department of Clinical Microbiology, Sahlgrenska University Hospital, Region Västra Götaland, Gothenburg, Sweden
6. Centre for Antibiotic Resistance Research (CARe), University of Gothenburg, Gothenburg, Sweden
7. Department of Paediatrics, Queen Silvia’s Children Hospital, Sahlgrenska University Hospital, Gothenburg, Sweden
8. Institute of Health and Care Sciences, University of Gothenburg/Sahlgrenska Academy
9. Faculty of Social and Health Sciences, Inland Norway, University of Applied Sciences, Elverum, Norway
10. Østfold University College, Halden, Norway
11. Faculty of Medicine, Public Health & Nursing, Universitas Gadjah Mada, Yogyakarta, Indonesia
12. School of Nursing and Midwifery, Griffith University, Gold Coast Queensland, Australia.

Corresponding author :

Jeannière T. Manegabe ([jeannieremanegabe6@gmail.com](mailto:jeannieremanegabe6@gmail.com))

ORCHID : 0000-0002-7149-7742

**ABSTRACT**

Antimicrobial resistance remains a pressing global challenge, driving efforts worldwide to reduce antibiotic consumption and mitigate its emergence and spread. The aim of this study was to describe how utilizing antibiotics for children under five years of age in the Democratic Republic of the Congo (DRC) is experienced by various healthcare providers. A phenomenographic approach was employed to analyse data from individual interviews with 14 healthcare workers of the Eastern DRC, including medical doctors, nurses and practicing pharmacists. Two overarching themes with their corresponding categories emerged: “Factors influencing antibiotic use” and “Operating within a weak healthcare system”. Healthcare providers consistently described widespread self-medication practices among children, driven largely by financial constraints within the population. Although many providers recognized the risks associated with inappropriate antibiotic use, their decisions were heavily influenced by systemic limitations and community expectations. A major concern raised was the increasing failure of antibiotics in treatment. This study highlights the complex factors shaping antibiotic use and prescription practices in Eastern DRC, where healthcare workers operate within a fragmented healthcare system. Addressing antibiotic resistance in such settings requires coordinated local and global efforts focused on promoting responsible antibiotic use, strengthening healthcare infrastructure, providing continuous education, establishing national guidelines, and improving community awareness and access to quality care.

**Keywords**: *Children, antibiotics, healthcare workers, Phenomenography, antimicrobial resistance*

**Introduction**

Antimicrobial resistance (AMR) is one of the foremost global public health and development threats. In 2019, bacterial AMR was directly responsible for an estimated 1.27 million deaths worldwide and contributed to nearly 5 million deaths. [1] AMR not only poses a significant health risk but also undermines the global economy. This underscores the urgent need for a global, coordinated action plan to address AMR, including financial and technical support for low- and middle-income countries (LMICs). [2–4]

Between 2000 and 2010, antibiotic consumption increased by 36% across 71 countries.[5,6] Antibiotics can be lifesaving, but all antibiotic use puts selective pressure on exposed bacteria, thus enabling rise of bacterial AMR. AMR affects countries across all regions and income levels, with its drivers and consequences exacerbated by poverty and inequality. LMICs are particularly impacted due to factors such as poor sanitation and infrastructure, facilitating the spread of resistant bacteria. [7] High prevalence of AMR has also been associated with contaminated potable water and poor water quality. [7–9]

Interviewed primary healthcare workers in Moshi, Tanzania, identified a wide variety of challenges in the management of children below five years of age, such as non-compliance to antibiotic treatment, self-prescription of antibiotics and lack of resources. [10] The health professionals also expressed a need for updated clinical guidelines and support. [10]

In Democratic Republic of the Congo (DRC), data on AMR are scarce. A high rate of antibiotic-resistant pneumococci were found in the nasopharynx of children aged one to five years in the general population of the Eastern DRC [11]. Other bacteria have been included in surveillance studies, such as *Salmonella* spp. and *Staphylococcus aureus* [12], but the prevalence of antibiotic resistance among other bacterial species are unknown in many parts of the country. [12] Hence, no national guidelines on empirical antibiotic treatment exists, leading local hospitals, universities or organisations to each create their own clinical practice or written guidelines [12]. Moreover, the healthcare system in DRC is fragmented with many different healthcare providers, including private practitioners and non-governmental organizations.

In 1933, the DRC enacted legislation regulating the prescription and sale of pharmaceutical products, restricting antibiotic prescription to qualified health personnel such as medical doctors and prohibiting the sale of antibiotics by non-pharmacists. Despite this, over-the-counter sales of antibiotics by unqualified personnel remain common, with antibiotics readily available in retail drug shops (commonly called “pharmacies”) and even through street vendors. [3,13,14] A recent survey of antibiotic dispensing at pharmacies (both authorised and unauthorised) in Kinshasa, DRC reveal a high use of ‘Watch’ antibiotics, as designated by the World Health Organization (WHO), which should be used selectively, such as third generation cephalosporins.[24] However, healthcare workers, including pharmacists, can play a crucial role in combating resistance by enhancing infection prevention and control measures, and ensuring antibiotics are prescribed and dispensed only when necessary. [15]

Although the DRC carries a high burden of both infections and AMR in the child population, little is known about the patterns of antibiotic use, the drivers of misuse, and their contribution to the growing problem of AMR, particularly in children under five, who are among the most vulnerable to infectious diseases. Persistent political instability in the DRC has further weakened the health systems, worsening vulnerabilities and complicating efforts to address AMR. The aim of this study was to describe the experiences of various healthcare providers regarding antibiotic use in children under five years in rural and urban areas of Bukavu, Eastern DRC.

**METHODS**

**Study design and approach**

This qualitative study adopted a phenomenographic approach collecting data through individual interviews, to explore the qualitatively different ways in which healthcare providers in Eastern DRC conceptualize and experience antibiotic prescribing for children under the age of five. Phenomenography was selected as the study’s methodological approach to explore the varied ways in which participants experience and understand antimicrobial use and access to care. This approach is particularly well suited to capturing a range of life-world perspectives within the region’s complex sociocultural, linguistic, and political landscape. Originally developed in the 1970s in educational research to examine differences in students’ learning experiences [16], phenomenography provides a well established framework for identifying qualitative variation in how individuals make sense of a shared phenomenon making it especially relevant to our study objectives.

**Study setting**

The research was conducted in Nyantende (rural) and Ibanda (urban) health zones, located in Bukavu, South Kivu province in eastern DRC. Bukavu has experienced rapid and unplanned urban expansion, particularly since the 1990s, largely driven by conflict-related displacement and rural-urban migration. This population growth has significantly strained urban infrastructure and health services, contributing to stark disparities in healthcare access between urban and peri-urban/rural areas. These contextual dynamics are critical for understanding healthcare-seeking behaviors and for implementing effective antimicrobial stewardship interventions in both settings. During the 1990s, population pressures intensified as a result of these dynamics. Notably, in 2015, South Kivu where Bukavu is located, recorded a very high under-five mortality rate, with nearly 15% of children not surviving to their fifth birthday [17,18]. Encouragingly, by 2023, this rate had decreased to approximately 5% [19], reflecting gradual improvements in child health outcomes despite ongoing systemic challenges.

The Nyantende Health zone, located 16 km from Bukavu, serves 132,000 inhabitants including 29,000 (22%) children. The average population density is 990 inhabitants per square kilometre. [20–22] The Ibanda Health zone, located in Bukavu City, serves 531,000 inhabitants including 101,000 (19%) children.

### Data preparation and collection

In preparation for data collection, the Congolese research team comprising faculty members from the Université Évangélique en Afrique participated in an intensive training workshop conducted in June 2022. The workshop was led by FM, ME, ML, and RM, with AMB contributing paediatric clinical insights. The training introduced the team to foundational principles of qualitative research and provided an in-depth orientation on the phenomenographic methodology, drawing on resources such as *The SAGE Encyclopedia of Qualitative Research Methods*. It also covered key elements of qualitative data collection and analysis, including coding techniques, ethical principles in research, and the importance of bracketing assumptions. Particular emphasis was placed on identifying experiential variation and interpreting meaning within specific social and cultural contexts.

To guide the interviews, a semi-structured interview guide was developed collaboratively by AMB, FM, and RM. The interview questions were carefully designed to explore healthcare providers’ experiences and perspectives on antibiotic use in children under the age of five. Topics included the length of time providers had been caring for children under five, common childhood illnesses treated in their facilities, frequently prescribed medications, views on antibiotic use, and professional advice regarding the appropriate use of antibiotics in paediatric care.

Data collection was carried out in June 2022 by the trained Congolese team alongside RM. In-depth interviews were conducted with healthcare professionals, including prescribers and pharmacists, working in urban healthcare centres, a rural district hospital, and private pharmacies across different sites in Bukavu. All participants gave written informed consent prior to the interviews, which were conducted using the collaboratively developed guide. Interviewers took notes and recorded responses to ensure accuracy and depth of information.

Despite language limitations which required the use of French, Swahili, and occasionally Lingala during both training and tool development, the research team made deliberate efforts to integrate local languages and culturally meaningful expressions into the data collection process. This attention to linguistic and cultural distinctions enhanced the contextual sensitivity of the study and promoted more authentic and meaningful engagement with participants. As the study progressed, the interview guide was iteratively refined to reflect emerging themes and to support a deeper exploration of evolving issues. This adaptive and reflective process allowed the researchers to capture collective patterns in healthcare providers’ prescribing practices, contributing valuable insights into antimicrobial stewardship in low-resource settings.

**Study tools**

The interview guide was developed by reviewing relevant literature and consulting with local healthcare experts to ensure it addressed key themes related to antibiotic prescribing practices. By validating and pre-testing the interview guide through pilot interviews with healthcare workers not included in the study, the questions could be refined for increased clarity and avoidance of potential bias. The guide was further reviewed by a panel of local health experts and qualitative researchers from University of Gothenburg to ensure cultural appropriateness and relevance. The prompts were open-ended and neutrally worded to minimize leading responses, and we actively used follow-up questions to ensure participants could freely share their perspectives. This process helped minimize bias and ensure the data reflected participants' genuine experiences.

**Participants**

In total, 14 healthcare workers participated in the study, comprising four medical doctors and four nurses from one district hospital (rural) and two health centres (urban), and six pharmacy providers from six pharmacies (three rural and three urban) (Table 1). All individuals working at the private pharmacies were nurses by training but are here referred to as pharmacists, to align with the local terminology. All the included pharmacies were authorised to sell drugs. Although not officially permitted, pharmacists in the DRC commonly prescribe antibiotics in practice. The healthcare facilities were chosen to represent different geographical and socioeconomic parts of South Kivu urban and rural district in DRC. The head of each facility was visited by a member of the team prior to data collection to inform about the aims of the study and to obtain approvals. All of the faith-based facilities approached agreed to participate, while one private facility declined. From each health centre with multiple prescribing clinicians on duty, 2–3 participants were included based on availability and educational backgrounds.

**Table 1: Characteristic of participants**

| Healthcare worker | Age  (years) | Sex | Educational background | Work experience | Health care facility | Area | Zone |
| --- | --- | --- | --- | --- | --- | --- | --- |
| 1 | 30-35 | M | MD | 12 years | HC | Urban | Ibanda |
| 2 | 30-35 | M | MD | 3 years | HC | Urban | Ibanda |
| 3 | 40-45 | M | MD | 5 years | GH | Rural | Nyantende |
| 4 | 30-35 | F | MD | 2 years | GH | Rural | Nyantende |
| 5 | 50-55 | M | Nurse | 17 years | HC | Urban | Ibanda |
| 6 | 25-30 | F | Nurse | 5 months | HC | Urban | Ibanda |
| 7 | 45-50 | M | Nurse | 10 years | GH | Rural | Nyantende |
| 8 | 40-45 | M | Nurse | 16 years | GH | Rural | Nyantende |
| 9 | 30-35 | F | Nurse/pharm | 7 years | P | Urban | Ibanda |
| 10 | 25-30 | M | Nurse/pharm | 5 months | P | Urban | Ibanda |
| 11 | 25-30 | F | Nurse/pharm | 5 years | P | Urban | Ibanda |
| 12 | 20-25 | F | Nurse/pharm | 3 years | P | Rural | Nyantende |
| 13 | 60-70 | M | Nurse/pharm | 40 years | P | Rural | Nyantende |
| 14 | 30-35 | F | Nurse/pharm | 3 months | P | Rural | Nyantende |

F: Female GH: General Hospital HC: Health Centre M: Male P: Private pharmacy MD: medical doctor Pharm: pharmacist (all pharmacists were nurses by education)

### Data Management and Analysis

The data collection process involved in-depth, one-on-one interviews with participants, each conducted at the participant’s workplace to ensure comfort and familiarity. These interviews lasted between 30 and 45 minutes, allowing for deep exploration of participants’ perspectives in a setting they were accustomed to. Language played a central role in facilitating open communication; interviews were conducted in Swahili, French, or a mix of both, and occasionally incorporated Lingala, depending on each participant’s preference and fluency.

This multilingual approach ensured inclusivity and created a safe space for participants to express themselves authentically. In addition to the interviews, researchers also undertook a visual inspection of pharmacies, examining the physical conditions and storage practices of medicines to supplement the narratives shared during interviews.

All interviews were digitally recorded using handheld audio recorders to ensure the fidelity of participants’ voices. These recordings were then transcribed verbatim to French by two members of the research team. Where necessary, the transcripts were translated into English with great care to preserve the voices of the original language. The translation process itself served as an additional layer of data familiarization, allowing researchers to engage more deeply with the content. Each transcript was then thoroughly reviewed by cross-checking it against the audio recordings to ensure accuracy and integrity of the data.

Given the resource-limited and politically fragile setting of this project, a manual thematic analysis approach was adopted at first. This decision was both pragmatic and purposeful: while qualitative software could have enhanced efficiency, it was not feasible for local researchers due to limited computer literacy and lack of reliable digital infrastructure. The manual process, however, offered a valuable learning experience and allowed for a more tactile and grounded engagement with the data.

The thematic analysis followed an iterative and reflective cycle. The process began with repeated readings of the transcripts followed by the generation of initial codes that captured key ideas, patterns, and sentiments expressed by participants. Color-coded pens and highlighters were used to manually tag segments of text, enabling the visual mapping of connections between codes. These initial codes were then grouped into broader, more abstract categories, and gradually, through ongoing comparison and reflection, a thematic map began to take shape.

Initial coding was led by the first author (JM), with input and validation from RM and AM. A second, independent coding was thereafter made by ME by using NVivo 14 software (QRS International). The emerging themes were discussed and refined collaboratively through regular team meetings including all co-authors, where insights were shared, and challenged interpretations, or any coding discrepancies, were resolved through open dialogue and consensus. This collaborative process not only enhanced the credibility of the analysis but also enriched the learning experience for the entire team.

**Ethics**

The study was approved by the Official University of Bukavu Research Ethics and Review Committee, Bukavu, DRC (No. 154/2022). All participants received both written and verbal information about the aims of the study and signed consent forms prior to the interviews.

**RESULTS**
The themes and categories represented different conceptions of healthcare providers’ experience of antibiotic use in children under five years of age (Table 2).

**Table 2. Summary themes and categories that emerged during data analysis**

|  | **Themes and categories** | **Citated in no. of interviews** |
| --- | --- | --- |
| **Theme 1.** | **Factors influencing antibiotic use** |  |
| 1.1 Category | Common or severe diseases in children | 14 |
| 1.2 Category | Limited knowledge among pharmacists or drug vendors | 4 |
| 1.3 Category | Self-medication of children is widespread | 14 |
| 1.4 Category | The effect of poverty on healthcare seeking behaviour | 6 |
| **Theme 2.** | **Operating within a weak healthcare system** |  |
| 2.1 Category | Dubious antibiotic supply chains | 8 |
| 2.2 Category | Handling treatment failures | 8 |
| 2.3 Category | Ambivalence when encountering shortcomings | 8 |

1. **FACTORS INFLUENCING ANTIBIOTIC USE**

The first theme describes the participants’ motivation for prescribing or initiating antibiotic treatment in children under-five, the widespread practice of caregivers administering medication to children without professional guidance and the effect of poverty on healthcare seeking behaviour.

- 1. **Common or severe diseases in children**

Diseases perceived as common by healthcare workers (HCWs) were similar in both urban and rural areas. Respiratory tract infections, gastroenteritis, malaria, and pneumonia were frequently mentioned, with malnutrition more often noted in rural settings. *"The types of infectious diseases that affect children are cases of gastroenteritis and pneumonia."* (HCW 1, medical doctor, urban) Malaria was mentioned by both urban and rural healthcare workers, whilst malnutrition was only mentioned by medical doctors in the rural area. “*The type of disease, the first unequivocally is malaria, then comes pneumonia and then the others, but both are more frequent, but recently we have had a lot of cases of malnutrition”* (HCW 3, medical doctor, rural)

Some pharmacists mentioned local names or practices for curing common illnesses. *“Malaria, diarrhoea, vomiting, and pharyngitis. For tonsillitis, there are those who arrive when they have already cleaned the throat manually (kugusa or kukoropa) and others treated with medicine.”* (HCW 14, pharmacist, rural)

Commonly used antibiotics were also similar in urban and rural areas. *“In the case of sepsis, we usually give ampicillin in combination with gentamicin’s and if there is resistance, we give cefotaxime.”* (HCW 7, nurse, rural) The use of third generation cephalosporins were common at healthcare facilities in both urban and rural areas. *“We use cefotaxime and ceftriaxone for respiratory infections because these antibiotics react (more) quickly for respiratory infections compared to ampicillin.”* (HCW 6, nurse, urban) Some pharmacists also related illness to certain antibiotics, although with a less targeted approach. *“Amoxicillin cures many illnesses, coughs for example and urinary tract infections, but most of the time amoxicillin is indicated for respiratory tract infections”* (HCW 10, pharmacist, urban)

One medical doctor mentioned a systematic use of antibiotics in children treated at the hospital, meanwhile showing awareness of established local guidelines.

*“We give antibiotics almost systematically for any case that arrives at the hospital (…), but also following the local guidelines for the treatment of severe malaria, we associate either macrolides or cyclins antibiotic, it is in this sense that we give antibiotics…”* (HCW 2, medical doctor, rural)

- 1. **Limited knowledge among pharmacists or drug vendors**

Limited scientific knowledge of the indication or the appropriate dosage of antibiotics was evident among some pharmacists. One pharmacist was not aware of the specific indications for the antibiotics he administered, nor the appropriate age for their use:

*“Yes yes, I give oral penicillin to children over 7–8 months but not before 7 months, I do not give because it is a drug that must be given with great caution because of its side effects. Overdose is not good. (…) Oral penicillin is given as a preventive antibiotic to treat internal wounds.”*
(HCW 10, pharmacist, urban)

Another urban pharmacist expressed a profit-driven approach, encouraging purchases of broad-spectrum antibiotics without adequately addressing potential risks:

*“I give antibiotics but not amoxicillin, which costs less (laughs). (…) I often give Augmentin (amoxicillin/clauvanic acid), cefixime or Cemycine (azithromycine) to treat malaria or angina (sore throat), which are frequent in children, and sometimes I combine two antibiotics with Efferalgan (paracetamol) if the fever persists.”* (HCW 11, pharmacist, urban)

The lack of awareness of the potential risks of overusing or overdosing antibiotics among some pharmacists or drug vendors, was echoed by a medical doctor, who associated this to limited training:

*“First of all, our population is an uneducated population, which means that there are a lot of antibiotics that are given, used out of order in the community by certain pharmacists who are not well educated or whoever. Someone who is taken to sell drugs, you will find that there are drugs he delivers to the patient which are not really good drugs or doses higher than the paediatric doses which are well recommended by (…) the protocols that are available.”* (HCW 4, medical doctor, rural)

- 1. **Self-medication of children is widespread**

Self-medication of children in the community, usually with antibiotics, were experienced by all participants as a widespread practice. This category comprises the healthcare workers conceptions of self-medication in children, perceived consequences and how they respond.

*“…the majority, if not 100% of parents self-medicate their children before bringing them to the hospital and when they notice that the child's condition is worse (…) I can say that all of them self-medicate their children before bringing them to the hospital either by antibiotics that they give abusively or by traditional medicines…”* (HCW 7, nurse, rural)

Self-medication was associated to the common experience of suspected or confirmed bacterial resistance.

*“…because by giving (antibiotics) out of order like that, we see a lot of cases of resistance. A mother arrives (…) you start a treatment, after a while you see that this treatment does not give good results, we do the culture, we isolate another germ which is resistant (…) after the lady will now be able to tell the truth that the child had already benefited from this treatment.”* (HCW 4, MD, rural)

Another potential consequence of self-medication in children was overdosing, leading to severe side-effects, as described by one rural medical doctor:

*“I don't know if this will surprise you, but we have already had cases of kidney failure in children eeehhh (laugh), when you search you will find that this is a child who has been given very strong antibiotics and has adult dosages, we have already had cases, many even, more than 10.”* (HCW 3, MD, rural)

The nurses and medical doctors had different strategies when encountering a child who had been given antibiotics before being brought to the healthcare facility. Some stopped the current treatment in favour of a diagnostic re-orientation, some used the same antibiotic at a higher dose and some immediately switched antibiotic. The following citation is an example of the second option:

“*For example, there are cases where they come with bottles of ampicillin and say that they were giving 250mg per day, at which point we tell them that we will continue with the same antibiotic but with a slightly higher dose.”* (HCW 8, nurse, rural)

In general, the pharmacist tried to mitigate the practice of self-medication by using their knowledge to determine if the ill child may be treated with drugs from the pharmacy, or if the child must be brought to hospital.

*“I can say that it is not at all advisable to use antibiotics first. But in our society, most parents buy drugs for children without consulting; others do it based on the advice of a neighbour who testifies that his child also suffered with the same symptoms when it should not be done like that because children can present the same sign, but they do not suffer the same sickness. The best thing is to come to the pharmacy, also give your complaint and buy the drugs offered by the pharmacist, also following the instructions given, if he tells you to go to the hospital then you must do it.”* (HCW 9, pharmacist, urban)

- 1. **The effect of poverty on healthcare seeking behaviour**

In this category, challenges associated with the economical backdrop of the community were observed. Most participants recognised that it was mainly financial constraints that caused low-income families to buy antibiotics for their children straight from the pharmacy, without first attending the healthcare facility.

“*Sometimes there are other patients who lack financial means; and do not have any possibility to be treated in the hospital (…) they come to see us to seek help because they trust our service.”* (HCW 9, pharmacist, urban)

Further, to avoid paying consultation fees, mothers could reuse a single antibiotic prescription for multiple children, confidently sharing her experience with other mothers.

*"Mums are experts in treating children and most of them start recommending the antibiotics that worked for their child to other mothers. Sometimes they keep the prescription for a long time and use it every time when the child gets sick. She uses the prescription to buy the same medicine even if the prescription expired. The same prescription can sometimes be used for all sick children in the family. I don't know if it's poverty that makes them think that the hospital is expensive to do this. In addition, they arrive at the hospital after having tried a lot of medicines at home…“* (HCW 5, nurse, urban)

One rural medical doctor had taken the initiative to teach mothers about the risks of self-medication and described the response given by the mothers, confirming that the driving factor behind self-medication were to avoid costs.

*“…when I discourage them from buying the drugs and give to the children, they say the hospital is expensive, she can't come to the hospital because she doesn't have the means. So, she tries (to self-medicate the child) first, if that succeeds, she is saving to buy the medicine from the hospital. That's the main reason they say: ‘Dr what you are saying we understand but it is that we have no money.’”* (HCW 3, MD, rural)

Lastly, low household income was reported to influence health-seeking behaviour, with many women opting for traditional treatment, as noted by healthcare workers. In this context, traditional treatment refers to care provided by traditional healers or herbalists who use indigenous knowledge, spiritual practices, and natural remedies, and are often perceived as more affordable and accessible than formal medical services. However, use of traditional medicine was also associated to possible harm to the exposed child.

*“The biggest challenge is when a child is brought to us and given antibiotics without knowing the dose; mixing antibiotics with traditional medicines can lead to the death of the child.”* (HCW 8, nurse, rural)

**2. OPERATING WITH A WEAK HEALTHCARE SYSTEM**

The second theme explored the challenges encountering the healthcare workers as they operate within a fragmented healthcare system among a poor population.

**2.1 Dubious antibiotic supply chains**

This category comprises healthcare workers experiences of differences in efficiency between antibiotics produced by different manufacturers and storage of antibiotics at pharmacies. Inadequate storage could have significant consequences, such as the degradation of antibiotics, especially those that are sensitive to temperature fluctuations. Nevertheless, an integral antibiotic supply chain is vital for efficacy of the drug in the end-user. One nurse opted for the government to intervene:

*“…we see the drugs effective or less effective depending on the manufacturing house (…) it is not understandable that for a product like ampicillin the quality varies according to the manufacturing house (…) The health zone forces itself to distribute more or less effective drugs, but many products that we buy from pharmacies are not effective, which is why I say that the government must get involved to resolve this situation.”* (HCW 5, nurse, urban)

Some pharmacists described how products produced in Europe were more efficient, and expensive, compared to the usual products they sold. Upon probing if there were any antibiotics they did not readily prescribe, one pharmacist responded:

*“Yes, it is for example Oroken (cefixime) (…) We cannot give the specialty (non-generic antibiotics) quickly (…) we use it if the first has shown resistance.”* (HCW 9, pharmacist, urban)

Storage of antibiotics emerged as a critical issue, with concerns about temperature control, accessibility, and protection from external elements. Many pharmacists reported not implementing proper storage practices due to limited resources, particularly the lack of refrigeration. As a result, pharmacists may be preferentially dispensing less shelf-stable antibiotics, which could compromise the effectiveness of treatment. In some cases, the lack of proper storage may limit the availability of certain antibiotics altogether, as those requiring refrigeration might not be stocked at all. One urban pharmacist described the situation:

*“We don’t have a fridge for the temperature, we put the drugs on the shelves or either we keep them in the boxes… I do everything to prevent dust from touching them!”* (HCW 10, pharmacist, urban)

Similarly, a rural pharmacist affirmed that antibiotics were stored in boxes and on shelves, without proper temperature control, which may affect the quality and availability of certain drugs. The lack of proper storage practices could therefore directly influence the selection and effectiveness of antibiotics dispensed to patients.

**2.2 Handling treatment failures**

Healthcare workers all faced challenges with treatment failures, which were often associated to incorrect use of antibiotics or suspected antibiotic resistance. One nurse gave this definition:

*“There is resistance when the antibiotic of choice is chosen and given correctly: respect of the dose, of the time, but after three days we notice that there is no improvement and that the signs still remain.”* (HCW 7, nurse, rural)

If the child did not improve despite antibiotic treatment, most healthcare workers described a stepwise escalation, starting with first-line treatments and switching to broader-spectrum antibiotics based in the child’s clinical response.

*"...if it's bronchitis, we start with ampicillin plus gentamicin and then for 48 hours, we see how the child is improving. If he doesn't improve, sometimes we upgraded to Augmentin* (amoxicillin/clavulanate acid)*, if Augmentin also does not respond we go up to ceftriaxone.”* (HCW 4, medical doctor, rural)

When pharmacists encountered treatment failure, they applied different strategies. Some checked whether the prescription had been respected, if not, the same treatment was repeated before changing the medicine. Others changed antibiotics directly to broader antibiotics or a different brand as described above. If an adjusted treatment failed, all pharmacists agreed that they would advise the patient to go to a healthcare facility for diagnose and treatment. This was illustrated in one interview when the pharmacist was probed what he would do in case a parent comes back due to treatment failure in a child:

*“I check to see if the instructions for use and the duration of the treatment have been respected and if I find that the cure has not been followed properly, I take the same treatment again and I advise you to follow the treatment.”* (HCW 10, pharmacist, urban)

*Upon further probing, assuming the parent comes back again, the pharmacist responded: “In this case, I change the antibiotic, giving cloxacillin for example, and if that fails, I send the patient to the clinic.”* (HCW 10, pharmacist, urban)

**2.3 Ambivalence when encountering shortcomings**

Whilst some healthcare workers expressed their views and experiences with much certainty, others expressed an ambivalence when encountering shortcomings within the weak healthcare system in which they operate. One medical doctor described how lack of means leads to very few diagnostic tests being performed and a possible misuse of antibiotics.

*“This is where we misuse antibiotics now because we have no tools, we try this or that. The few patients who have the means, we send them to Panzi (general hospital) or elsewhere to do the antibiogram, the blood culture to have the results. Otherwise, in the majority of cases, we give presumptive antibiotic therapy.”* (HCW 3, medical doctor, rural)

Further, another medical doctor described upon probing how healthcare staff also contribute to antibiotic misuse, habits performed despite better knowledge.

*“…look, even when a patient comes to hospital and you don't prescribe him any medication, he thinks that you haven't treated him. (…) it's even more serious for the nursing staff because they start by treating the patient on their own if they have any doubts, they take them to hospital and then, either you can tell them that the check-up shows nothing, and they (should) just give paracetamol, they'll tell you to give them an antibiotic themselves. It's lovely… It has become our characteristic and maybe out of fantasy we do it, we know it's not good but we do it.”* (HCW3, medical doctor, rural)

A pharmacist described how her background in nursing meant she needed to respond to the needs of a patient, even though they did not go for the advised diagnostic tests.

*“Before taking a drug, someone must undergo laboratory tests. (…) And we give them this advice, but they don't accept it. As a nurse, you must save the emergency first, you must treat him first. (…) But if he doesn't go for the tests and he's in an emergency, you treat him first, waiting to see if he goes for the test, or if another day he falls ill, he'll go for the tests, but at least you've given him the advice.”* (HCW 14, pharmacist, rural)

Another pharmacist encountered parents who only bought part of an antibiotic course due to lack of means, or who did not finish the child’s antibiotic course. This leads to a resignation in his own power to change the situation.

*“…it's the money they don't have. It's like the one who came to ask me to give him the medicine for 100 francs, 200, francs, 300 francs. Many don't buy the whole dose. They buy one tablet, and we tell them it's no good, but what can we do? (…) they don't finish the cure (in the child). It's the ones from the city, from your place, who finish the cure. Here, it's God who helps us. We heal by God's grace.”* (HCW 13, pharmacist, rural)

**DISCUSSION**

This study explored the factors influencing antibiotic prescription among medical doctors, nurses and pharmacists in both urban and rural areas of the Eastern DRC. The interviews revealed a complex interplay between clinical decision making, patient related factors and healthcare limitations. The findings highlight the widespread use of antibiotics among children in the community, common experiences of treatment failure that could be due to poor quality of antibiotics or antibiotic resistance, and lastly, how in general healthcare workers of all professions tried to mitigate the shortcomings of the healthcare system.

All healthcare workers, including the pharmacists, interviewed in this study had either been approached in an informal way by mothers expecting a prescription for antibiotics for their child, or had encountered children in whom antibiotic treatment had been initiated at home. Similar practices was recently observed in a qualitative study in Moshi, Northern Tanzania [10] and could be explained by low socioeconomic level of the population in both studies. As highlighted in the study from Tanzania, mothers avoid hospital visits due to fear of costs, including consultation fees, laboratory fees and other hidden costs. The healthcare system in the DRC does not subsidize healthcare visits for children under five, meaning that mothers are expected to pay 5 or 10 USD for each visit. As a result, women in Eastern DRC may become their own “experts,” as described by one nurse in this study, often reusing previous prescriptions for other children or for new episodes of illness. These self-medication practices are driven by the financial barriers that limit access to formal healthcare services and highlights the coping strategies employed by mothers in the absence of affordable healthcare options.

Most countries have legal regulations that restrict the sale of antibiotics without prescription, but in LMICs these are often poorly enforced due to limited funding and weak healthcare systems. However, underenforcement may also be part of a semi-official strategy to support healthcare access for populations with limited access to government health services. Thus, purchasing antibiotics from pharmacies can be considered a rational strategy to cope with the chronic issue of difficult access to health services.[23] Further, the wider socioeconomic conditions in LMICs influence antibiotic use through several routes. Poor living conditions, including crowding and lack of sanitation, affect the majority of LMIC populations. This contributes to high illness rates, including high rates of infections. Antibiotics are in this setting frequently prescribed “just in case” [35], which was also observed in this study. The reasons for such prophylactic antimicrobial prescribing are known to be complex but can be understood as an extension of infection prevention and control measures. [36] The challenges identified in this study highlight the broader structural and socioeconomic factors influencing antibiotic use and the need for tailored interventions to address both healthcare practices and community behaviours.

This study revealed that healthcare providers, including nurses, pharmacists, and doctors, employed diverse approaches when prescribing antibiotics, often influenced by a combination of patient-related factors, limited resources, and informal consultations. These findings are consistent with patterns of antibiotic prescription behavior observed in other low-resource settings. The reliance on informal consultations is further exacerbated by the absence of clear national guidelines on antibiotic use, as seen in the DRC, where local institutions are left to develop their own protocols without sufficient data on local resistance patterns.

Moreover, some healthcare workers reported inefficiency of certain antibiotics, associated to the manufacturer or pharmacist/drug vendor. Although the qualitative nature of this study cannot confirm these conclusions, the prevalence of substandard or falsified drugs is a growing global issue [25]. A recent review on the prevalence of substandard, falsified or unlicensed drugs in Africa found that 23 % of pooled samples were substandard or falsified, with antibiotics accounting for 16 % [26]. Another study in southeast DRC aimed to validate an analytical tool to ensure the quality of sold in formal and informal pharmaceutical markets, also gave proof of a high prevalence of substandard antibiotics in DRC, particularly in the informal market (54 % vs. 11 %) [27].

The alarming prevalence of substandard/falsified antibiotics in DRC, especially in informal or unauthorised markets, requires urgent attention as it contributes to increased antibiotic resistance and a high death toll, particularly among children. [28] Additionally, the frequent use of broad-spectrum antibiotics in the community of DRC, revealed by the recent survey from Kinshasa [24] was echoed in some of the interviews in this qualitative study. Together with the frequent reports of treatment failures due to suspected or confirmed bacterial resistance, these findings underscore the critical need for comprehensive antibiotic surveillance in the DRC.

Limited scientific knowledge among some pharmacists and other healthcare providers contributed to misconceptions about antibiotics, including indications and dosages. Physicians’ knowledge and education are vital in shaping their antibiotic prescription practices. While the commitment to good clinical practice was evident among medical doctors and nurses in our study, the challenges within the management of antibiotics persist. To improve prescribing behaviour, it is crucial to gain a comprehensive understanding of antibiotic prescribing practices and the factors influencing this behaviour. One of the main theories that help comprehend physicians’ antibiotic prescription behaviour is the Teixeira antibiotic prescription behavioural model. This model builds upon the widely accepted knowledge-attitude-practices theory and incorporates considerations of external determinants that impact antibiotic prescription practices. [29,34] Previous studies have shown that continuing medical education activities and seminars, which provide training on updated antibiotic guidelines and information about antibiotic resistance, have a positive impact on physicians’ antibiotic prescription behaviour[29]. However, the quality of healthcare professionals remains a concern, with issues ranging from inadequate training facilities to underqualified teachers and outdated teaching programs. This was also observed by the DRC Ministry of Health. [21] Thus, challenges exist in ensuring access to quality guidelines, continuous education, and structural support. **Nevertheless,** addressing these challenges is essential to improving antibiotics prescription practices and overall healthcare quality [20].

A notable aspect of this study was the designation of nurses working in private pharmacies as pharmacists, despite their training being solely in nursing. This classification raises important considerations regarding the interpretation of the findings. While there is no definitive right or wrong approach to this designation, the absence of formally trained pharmacists could be considered a limitation. It is possible that the perspectives shared by these nurses, acting as pharmacists, were influenced by their nursing background, potentially shaping their approach to antibiotic dispensation in ways distinct from those of trained pharmacists. Buchan and Calman,[30] discuss the impact of nurse prescribing on healthcare delivery, suggesting that nurses may approach medication management differently based on their background. Similarly, Maier and Aiken [32] explore the expanding roles of nurses in primary care, noting that their expanded responsibilities can affect their clinical decision-making. In our study, the practicing pharmacists frequently described how they mitigate the shortcomings of the healthcare system by prescribing antibiotics based on symptoms to children whose family could not afford healthcare, or by recommending customers to seek healthcare when encountering treatment failure. This may be influenced by their background in nursing and by the fact that they were all operating in authorised pharmacies. It is likely that the including of non-authorised pharmacies (or drug vendors) with untrained personnel may have yielded very different results with the potential of more profit-driven approaches.

Despite shortcomings in the healthcare system and ambivalence in how to operate within it, healthcare workers in this study opted for responsible antibiotic use, proper healthcare-seeking behaviour, and the importance of accurate diagnosis before antibiotic initiation. The discrepancy in the use of antibiotics in Bukavu in the Eastern part of the DRC can be explained by the fact that Bukavu is a zone of conflict with a weak infrastructure and poor population. The DRC faces significant challenges in healthcare delivery, particularly in conflict-affected regions like Bukavu. The ongoing conflicts have led to widespread displacement, with over 1.7 million people displaced in the North Kivu province alone.

**Study Limitations**

This study has several limitations that warrant consideration. First, this was a qualitative study with a relatively small sample size, which limits the generalizability of the findings beyond the study context. In addition, the data collection process presented linguistic challenges: interviews were conducted in a mix of French, Swahili, and sometimes Lingala, then transcribed into French and later to English. This may have resulted in the loss or alteration of distinct meanings during the translation process. Second, the absence of patient perspectives reduces the comprehensiveness of the study, particularly in understanding the broader implications of healthcare service delivery.

Despite these limitations, this study contributes valuable insights by documenting the experiences of healthcare workers operating in a politically unstable and poor resources setting. These voices are often overlooked in research but are critical to understanding the realities of health systems under pressure.

**CONCLUSIONS AND RECOMMENDATIONS**

This study illuminates the challenges and factors influencing antibiotic use and prescription practices in children under five in the DRC, with a particular focus on the conflict-affected and resource-limited Eastern region. Despite the study's limited sample size (n=14), it offers valuable insights into a fragmented healthcare system. The findings highlight the pressing need for coordinated interventions, continuous education, national guidelines, and enhanced healthcare infrastructure to combat antimicrobial resistance. Addressing this issue requires unified efforts at both local and global levels to promote responsible antibiotic use and ensure effective stewardship.

In light of the challenges identified, such as informal consultations and financial constraints, integrating pharmacists more effectively into antibiotic stewardship efforts could improve prescribing practices. Given their expertise solutions, in medical management and antimicrobial resistance prevention, trained pharmacists should lead stewardship interventions, supported by nurses and pharmacy providers who can reinforce prescribing guidelines and patient education. Potential solutions such as referral and collaborative incentives could be explored to strengthen meaningful partnerships among healthcare providers, ensuring that pharmacists play an active and informed role in antibiotic prescribed decisions, with clear pathways for support from other healthcare cadres.

In addition, subsidized diagnostic testing could alleviate the financial burden on both patients and healthcare providers, enabling more accurate diagnoses and promoting the appropriate use of antibiotics. Together with the integration of the WHO AWARE framework, these strategies could help address existing barriers, promote stronger collaboration among healthcare workers, and ultimately enhance antibiotic prescribing practices and improve patient outcomes strategies could help mitigate the current barriers, enhance collaboration among healthcare workers, and ultimately improve antibiotic prescribing practices and patient outcomes.

**Acknowledgment**

We express our gratitude to all the healthcare workers who despite their many duties and demands took time to participate in the study. Sincere thanks to the Université Evangelique en Afrique and the Head of the University Ngongo Fatuma, PhD who provided and participated significant insights for the team conducting the interviews concerning antibiotic use in common childhood illnesses.

**Authors’ contributions**

The initial planning of the study was performed by RM, FM, BM, RA, SS and ME. The study was further designed by RM, FM and AMB with methodological advice from RA, SS, ML and ME. The interviews were recorded and transcribed verbatim by a member of the team (DS, GMK, JTM, JM and PK). ABM transcribed the interviews. JTM was mainly responsible for data analysis and writing. Discussion on themes and categories, and revisions of manuscript were performed by all authors. All authors read and approved the final manuscript.

**Funding**

The study was funded by the Centre for Antibiotic Resistance Research (CARe), University of Gothenburg, Sweden.

**Availability of data and materials**

The datasets generated and analysed during the current study are not available due to confidentiality clauses, but anonymised versions are available from the corresponding author upon reasonable request.

**Declarations**

**Ethics approval and consent to participate**

The study was approved by the Official University of Bukavu Research Ethics and Review Committee in Bukavu, DRC (No 154/2022). All participants received both written and verbal information about the aims of the study and signed consent forms prior to the interviews.

**Consent for publication**

Not applicable.

**Competing interests**

The authors declare that they have no competing interests.

**Reference**

1. Global burden of bacterial antimicrobial resistance in 2019: a systematic analysis. Lancet. 2022;399(10325):629-55.

2. Chan M. Global action plan on antimicrobial resistance. World Health Organisation. 2017;1–28.

3. Shankar Pr. Book review: Tackling drug-resistant infections globally. Arch Pharm Pract. 2016;7(3):110.

4. Tufa TB, Regassa F, Amenu K, Stegeman JA, Hogeveen H. Livestock producers’ knowledge, attitude, and behavior (KAB) regarding antimicrobial use in Ethiopia. Front Vet Sci. 2023;10:1–20.

5. Klein EY, Van Boeckel TP, Martinez EM, Pant S, Gandra S, Levin SA, et al. Global increase and geographic convergence in antibiotic consumption between 2000 and 2015. Proc Natl Acad Sci U S A. 2018;115(15):E3463–E3470.

6. Global antibiotic consumption 2000 to 2010: an analysis of national pharmaceutical sales data - The Lancet Infectious Diseases. https://www.thelancet.com/journals/laninf/article/PIIS1473-3099(14)70780-7/fulltext.

7. Collignon P, Beggs JJ, Walsh TR, Gandra S, Laxminarayan R. Anthropological and socioeconomic factors contributing to global antimicrobial resistance: a univariate and multivariable analysis. Lancet Planet Heal. 2018;2(9):e398–e405.

8. Finley RL, Collignon P, Larsson DGJ, Mcewen SA, Li XZ, Gaze WH, et al. The scourge of antibiotic resistance: The important role of the environment. Clin Infect Dis. 2013;57(5):704–710.

9. Société Française de, Pédiatrie. Guide de prescription d’antibiotique en pédiatrie. Arch Pediatr. 2016;23(3):1–55.

10. Emgård M, Mwangi R, Mayo C, Mshana E, Nkini G, Andersson R, et al. Tanzanian primary healthcare workers’ experiences of antibiotic prescription and understanding of antibiotic resistance in common childhood infections: a qualitative phenomenographic study. Antimicrob Resist Infect Control. 2021;10(1):1–15.

11. Birindwa AM, Emgård M, Nordén R, Samuelsson E, Geravandi S, Gonzales-Siles L, et al. High rate of antibiotic resistance among pneumococci carried by healthy children in the eastern part of the Democratic Republic of the Congo. BMC Pediatr. 2018;18(1):1–12.

12. Lupande-mwenebitu D, Alexandra S, Zakaria L, Lunguya-metila O, Lavigne J, Rolain J. Journal of Global Antimicrobial Resistance Current status of resistance to antibiotics in the Democratic Republic of the Congo : A review. Integr Med Res. 2020;22:818–825.

13. Eibs T, Koscalova A, Nair M, Grohma P, Kohler G, Bakhit RG, et al. Qualitative study of antibiotic prescription patterns and associated drivers in Sudan, Guinea-Bissau, Central African Republic and Democratic Republic of Congo. BMJ Open. 2020;10(9):1–12.

14. Ministère de la santé RC. Profil Pharmaceutique De La Republiquue Democratique Du Congo 2011. 2011;1–32.

15. World Health Organization. WHO’s first global report on antibiotic resistance reveals serious, worldwide threat to public health. World Health Organization. 2014;News Release.

16. Limberg LB. Phenomenography. In: Given LM, editor. *The SAGE Encyclopedia of Qualitative Research Methods*. 2012. Thousand Oaks. SAGE Publications, Inc: 612–614.

17. Aymar N, Mushaglusa M. Bukavu: etude exploiratoire sur la ville. Tom Herdt (Institute Dev Policy, Anvers) juin. 2021;9.

18. Ministère du plan et de la Révolution de la modernité RD du C. Institut national de la statistique: Profil de la province du Sud Kivu. doi:10.1787/9789264241763-131-fr.

19. ICF R-IN de la S et l’Ecole de SP de K et. Enquête Démographique et de Santé 2023–24 : Rapport des indicateurs clés. . 2024. Kinshasa, RDC et Rockville,Maryland,USA.

20. Sante M de la. Deuxième enquête démographique et de santé (eds-rdc ii 2013-2014). 2014. Rockville, Maryland, USA.

21. Republique Democratique du Congo Ministere de la Santé et Hygene Publique. Plan National De Developpement Sanitaire 2016-2020. Minist la Sante Publique. 2016;1–96.

22. Birindwa AM, Manegabe JT, Mindja A, Nordén R, Andersson R, Skovbjerg S. Decreased number of hospitalized children with severe acute lower respiratory infection after introduction of the pneumococcal conjugate vaccine in the eastern democratic republic of the congo. Pan Afr Med J. 2020;37(211):1–15.

23. Cabral C, Zhang T, Oliver I, Little P, Yardley L, Lambert H. JAC- Antimicrobial Resistance Influences on use of antibiotics without prescription by the public in low- and middle-income countries : a systematic review and synthesis. JAC-Antimicrobial Resist. 2024;6(5):1–21.

24. Kakumba JM, Kindenge JM, Kapepula PM, Iyamba JML, Mashi ML, Mulwahali JW, et al. Evaluation of Antibiotic Prescribing Pattern Using WHO Access, Watch and Reserve Classification in Kinshasa, Democratic Republic of Congo. Antibiotics. 2023;12(8):1–15.

25. WHO. A study on the public health and socioeconomic impact of substandard and falsified medical products. 2019 https://www.who.int/publications/i/item/9789241513432 Accessed 28th April 2025.

26. Asrade Mekonnen B, Getie Yizengaw M, Chanie Worku M. Prevalence of substandard, falsified, unlicensed and unregistered medicine and its associated factors in Africa: a systematic review. J Pharm Policy Pract. 2024;17(1):1–20.

27. Tshilumba PM, Ilangala AB, Kindenge JM, Kasongo IM, Kikunda G, Rongorongo E, et al. Detection of Substandard and Falsified Antibiotics Sold in the Democratic Republic of the Congo Using Validated HPLC and UV-Visible Spectrophotometric Methods. Am J Trop Med Hyg. 2023;109(2):480–488.

28. Nayyar GML, Breman JG, Mackey TK, Clark JP, Hajjou M, Littrell M, et al. Falsified and substandard drugs: Stopping the pandemic. Am J Trop Med Hyg. 2019;100(5):1058–1065.

29. AbdEl-Aty MA, Amin MT, Ahmed SM, Elsedfy GO, El-Gazzar AF. Exploring factors for antibiotic over-prescription in children with acute upper respiratory tract infections in Assiut, Egypt: a qualitative study. Antimicrob Resist Infect Control. 2024;13(1):1–16.

30. James Buchan and Lynn Calman. Skill-Mix and policy change in the health workforce: Nurses in advanced roles. OCDE Heal Work Pap. 2010;(54):17.

31. Reali S, Kwang YC, Cho JG, Alffenaar JW, Aslani P. Factors influencing physicians’ antimicrobial prescribing decisions: A systematic review of qualitative studies. Br J Clin Pharmacol. 2025;(April 2024):1330–1351.

32. Maier CB, Aiken LH. Expanding clinical roles for nurses to realign the global health workforce with population needs: A commentary. Isr J Health Policy Res. 2016;5(1):2–5.

33. Happell, B., Scott, D., & Platania-Phung C (2012). Perceptions of barriers to physical health care for people with serious mental illness: A review of the international literature. Issues Ment Health Nurs. 2012;33:752–761.

34. Teixeira Rodrigues A, Roque F, Falcão A, Figueiras A, Herdeiro MT. Understanding physician antibiotic prescribing behaviour: A systematic review of qualitative studies. International Journal of Antimicrobial Agents. 2013;41(3):203–212.

35. O’Neill J. Government response to the Review on Antimicrobial Resistance September 2016. Public Heal Engl. 2016;(September):33.

36. Denyer Willis L, Chandler C. Quick fix for care, productivity, hygiene and inequality: Reframing the entrenched problem of antibiotic overuse. BMJ Case Rep. 2019;4(4):1–6.
